# Hydroxyapatite Block Produced by Sponge Replica Method: Mechanical, Clinical and Histologic Observations

**DOI:** 10.3390/ma12193079

**Published:** 2019-09-21

**Authors:** Antonio Scarano, Felice Lorusso, Pablo Santos de Oliveira, Sanosh Kunjalukkal Padmanabhan, Antonio Licciulli

**Affiliations:** 1 Department of Medical, Oral and Biotechnological Sciences, University of Chieti-Pescara, Via dei Vestini, 31, 66100 Chieti, Italy; drlorussofelice@gmail.com; 2 Department of Oral Implantology, Dental Research Division, College Ingà, UNINGÁ, Cachoeiro de Itapemirim 29312, Brazil; psoliveiraodonto@yahoo.com.br; 3 Department of Engineering for Innovation, University of Salento, 73100 Lecce, Italy; sanosh2001@gmail.com (S.K.P.); antonio.licciulli@unisalento.it (A.L.)

**Keywords:** histology, sponge replica, scaffold, cone beam computed tomography, hydroxyapatite

## Abstract

Purpose: The grafting procedure for the anthropic ridges of jaws represents a surgical technique for increasing the bone volume to permit the placement of dental implants for oral rehabilitations. The aim of this study was to evaluate a hydroxyapatite (HA) porous scaffold produced via a sponge replica method for the treatment of maxillary bone defects in a human model. Methods: A total of thirteen patients were treated for sinus lifting in the posterior maxilla for a total of 16 defects treated with cylindrical HA Block. The experimental sites were evaluated by a 3D Cone Beam Computer Tomography scan (CBCT), and the histological analysis was performed after 3 months of healing. Results: After the 3 months healing period, the histological outcome of the investigation showed a high level of biological osteoconduction of the HA. Microscopical evidence of new bone formation was also observed in the central portion of the graft block. The samples were composed of different tissues: 39 ± 1% new bone, 42 ± 3% marrow space, 17 ± 3% residual HA Block and 4.02 ± 2% osteoid tissue were present. The new bone formation in the block was 8 ± 3%**.** Conclusions: The study findings support that HA porous scaffolds produced by sponge replica were effective for the treatment of maxillary bone defects in humans.

## 1. Introduction

The regeneration of extended and complex defects of the maxilla or mandible alveolar bone is a frequent requirement prior to implant treatment [1,2]. Different biological and synthetic bone substitute biomaterials have been proposed for the regeneration of bone defects [3]. The properties of the bone substitute material are a crucial factor that determines the success of the bone to bone repair. The grafting materials used in clinical practice were: autologous bone, calcium sulfate, bioglass, coralline calcium carbonate, polylactide-polyglicolide materials, hydroxyapatite, mineralized and demineralized freeze-dried allogeneic bone grafts, anorganic bovine, and synthetic polymers. Current bone regeneration includes the use of block grafts or particulate biomaterials with or without a barrier membrane [3,4,5,6,7,8,9]. An autogenous bone graft is considered ideal, a limited availability of materials from the intraoral donor site and the severe pain at the bone graft donor site, such as infection or morbidity and haemorrhage, make it challenging [10,11]. To date, many calcium phosphate-based grafts have been used in bone healing, and regeneration comes in a variety of resorption rates, chemical forms and shapes, such as particulate and block [12,13]. 

Among them, the hydroxyapatite-as-bone substitute has received strong interest due to its mechanical proprieties and space making, and it has already been used to repair various types of tissue defects. Hydroxyapatite (HA) is a synthetic bioactive material that has osteoconductive and osteoinductive properties. The inorganic component of bone tissue itself is made of a globular and plate structure distributed among collagen fibrils. For this reason, there is an increasing interest in the development of HA biomaterials as bone substitutes [14,15]. The new bone formed around the granules or inside the hydroxyapatite block is influenced by chemical compositions and dimensions.

In an attempt to avoid autologous bone harvesting, HA porous scaffolds produced via a sponge replica method were used in this study for filled standard cylindrical and experimental bone defects in humans. 

The purpose of this study was to perform, on human subjects, the histological and morphometrical evaluation of biopsies retrieved from bone defects filled with hydroxyapatite block obtained by a sponge replica method, after 3 months of healing (Sigma Aldrich, St. Louis, MO, USA).

## 2. Materials and Methods

### 2.1. Fabrication of HA Scaffold

HA porous scaffolds for a vertical bone augmentation with a desired shape and dimensions were obtained by the sponge replica method [12]. A reactive submicron powder was first synthesized by the sol–gel route with hydroxide coprecipitation using Ca(OH)_2_ (Sigma Aldrich, St. Louis, MO, USA) and phosphoric acid (Sigma Aldrich) as precursors. The as–synthesized powder was calcined in air at 900 °C for 60 min before use. The slurry for the sponge impregnation was prepared by adding the HA powder (70 wt%) within a 2 wt% Polyvinyl Alcohol solution (PVA). An organic deflocculating agent, Dolapix CE-64 (Zschimmer & Schwarz, Lahnstein, Germany), was used to obtain the optimal slurry viscosity for the impregnation. Small rectangular shaped polyurethane sponges (density of 30 kg/m^3^, 25 ppi) were impregnated with the HA slurry to get the desired HA scaffold structures. After infiltration, the sponges were then gently squeezed to remove the excess slurry and dried for 24 h in air. To get the final HA scaffold, the dried sponges were first heat treated at 500 °C at a slow rate of 1 °C/min to completely remove the polyurethane foam without collapsing the structure, and finally the samples were sintered at 1300 °C for 3 h. The polyurethane sponge dimensions were slightly bigger than those of the final desired HA substitutes to compensate for the sintering shrinkage.

### 2.2. Packaging and Sterilization 

In the present study, biomaterials with possible transmissions of iatrogenic diseases were not used; furthermore, according to the Spaulding classification, based on infective risk an adequate protocol of sterilization was followed [16]. The sterilization of the HA blocks was done by exposure to dry heat at 160 °C for 2 h. After sterilization, the scaffolds were packaged in the cleanroom. 

### 2.3. Device Production Quality Assessment

The preparation of the HA scaffold followed the quality conformity standard assessment and traceability system based on a unique identification number of the device, specifications for raw materials, labels, packaging processes, production methods of the intermediate and finished materials; device drawings, software design specifications, work instructions, environmental specifications, sterilization phases, inspective processes, acceptance criteria and validation, according to the legislation in force.

## 3. Characterization 

Scanning electron microscopy was used to characterize the pore morphology and microstructure of the scaffold, and X-ray diffraction analysis was used to study the phase and composition of the sintered scaffold. The initial and final dimensions of the scaffold were used to calculate the linear shrinkage (δ) of the samples during sintering. From the ratio between the weight and the volume of the scaffold, the bulk density (ρ) and finally the porosity of the bone substitute were calculated by the using the following equation:

P(%) = (1 − ρ/ρ_0_) × 100 (1)

where ρ is the bulk density and ρ_0_ is the apparent density of the dense material.

**Figure 1:**
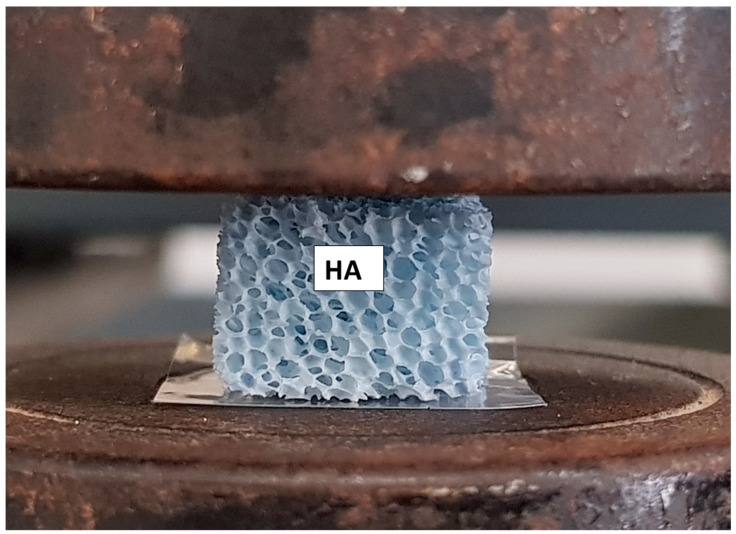
The HA scaffold during the mechanical properties test (HA).

The mechanical properties of the scaffold were evaluated with compressive tests on scaffolds having dimensions of 15 × 10 × 10 mm^3^. The samples were tested using a universal testing machine (Lloyd LR5K instrument, Berwyn, PA, USA) equipped with a 1 kN load cell at a crosshead speed of 0.5 mm/min (Figure 1). Five samples were tested to get a strength average value and a standard deviation.

### 3.1. In Vivo Experiment

The in vivo investigation obtained ethical approval from the Inter Institutional Ethics Committee of Faculdade Ingá, UNINGÁ, PR, Brazil, N 89018318.2.0000.5220, and the research was conducted in the Outpatient Department of Oral Implantology, Center for Advanced Studies, Dental Research Division, UNINGÁ-Cachoeiro de Itapemirim, Brazil, according to the Helsinki Declaration and Good Clinical Practice guidelines.

A total of 13 subjects without relevant past medical anamnesis (8 women and 5 men, mean age 55 years, range 41–64 years, all non-smokers,) were included for surgery; ten patients were treated for a unilateral procedure, and three subjects received a bilateral surgery, for a total of 16 maxillary bone defects.

The subjects were selected for a sinus augmentation procedure in order to receive dental implants into the posterior maxillary region. The surgical procedure was performed.

The inclusion criteria of the study were:

1. fully or partially edentulous/unilateral or bilateral loss of maxillary premolar with residual height of the alveolar ridge between 3 and 4 mm.

The exclusion criteria of the study were:

2. severe illnesses or uncontrolled diabetes;
3. neck and head radiation therapy;
4. radiotherapy or chemotherapy;
5. presence of a residual root, sinus pathology, periodontal disease;
6. tabagism.

All candidates underwent a preliminary examination at the first visit by Orthopantomography (OPT) radiographs.

A preoperative tomographical evaluation of the clinical case was performed by a three-dimensional Cone Beam Computed Tomography scan (CBCT) (Vatech Ipax 3D PCH-6500, Fort Lee, NJ, USA) taken to perform the surgical planning of the clinical case, to evaluate the patency of the ostium and the osteomeatal complex and to exclude evidences of sinus pathologies such as Schneider thickening, odontogenic sinusitis, allergies or cysts, complete to partial sinus obliteration, oroantral, mucoceles, mucopyoceles communications and antroliths.

Before surgery, the subjects were extensively informed on the surgical procedures of sinus grafting and implant insertion.

Six days before the surgery, the patients followed a professional hygiene prophylaxis session and were instructed about the correct domiciliary oral care. A prescription of 0.2% chlorhexidine digluconate solution (Curaden Healthcare S.p.A., Saronno, Italy) mouth rinsing for 2 min once a day was performed.

The anesthesia of the region was performed by the local infiltration of Articaine (Pierrel S.p.A, Milan, Italy) with epinephrine (1:100.000). According to Scarano et al., the surgical access was performed by a modified full thickness triangular flap without an anterior release incision [17]. An anthrostomy of the lateral bone wall was performed with an ultrasonic device (Variosurgery3, NSK, Tokio, Japan), with a cooling irrigation of sterile saline solution (5–6 °C) with a mesio-distal distance of 6 mm and a height of 6 mm in the apico-coronal orientation; then, the bony door was removed from the site. The Schneiderian membrane was gently detached, and the sinus was treated with a bone lamina technique without biomaterials [18]. 

**Figure 2:**
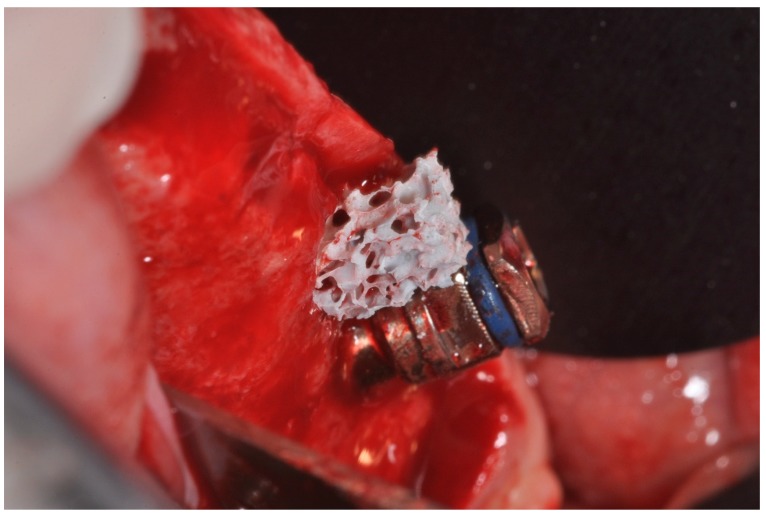
HA scaffold during placement in bone defect.

**Figure 3:**
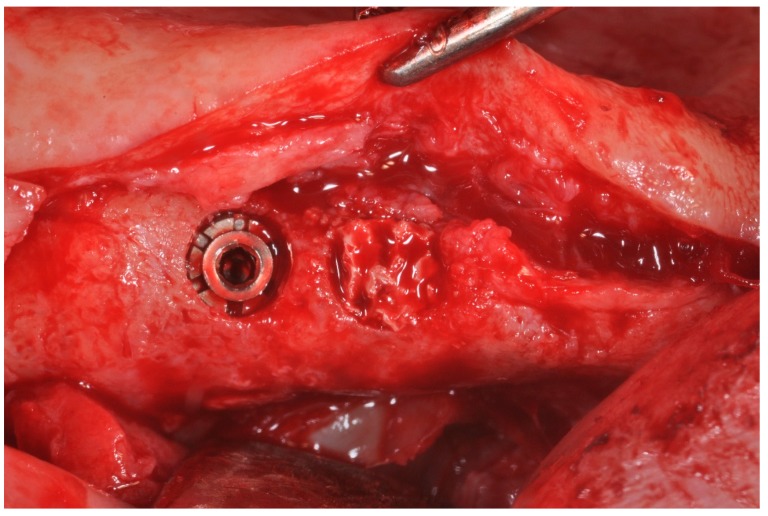
HA scaffold placement in bone defect.

The residual bone defect was filled with a cylindrical HA Block (Figure 2 and Figure 3). 

A total of 28 cylindrical implants (Isomed Albignasego, Padova, Italy) were placed in the treated sinuses. After the implant site drilling and dental fixture positioning, the flaps were carefully repositioned and sutured through the use of a black Poliamide 4.0 (Assut Europe, Magliano de’ Marsi, Italy). After a healing period of about three to four months, a screw-healing abutment was placed. After about four months, for the evaluation of the bone healing, bone cores were harvested in the central portion of the alveolar ridge of the maxillary, using a 4 mm diameter trephine drill irrigated and cooled via a sterile saline solution. At this time, new bone cores were harvested in the bone defect previously filled with the HA block, using a 4 mm diameter trephine drill cooled with a sterile saline solution irrigation (5–6 °C).

### 3.2. Processing of Specimens

The bone core biopsies were fixated in a buffered formalin solution (10%) and processed for histomorphometry and histology with thin ground sections with the Scan 1 Automated System (Assing, Pescara, Italy) [19]. The bone samples were dehydrated in an ascending rinses series of alcohol solutions and included in a glycolmethacrylate resin (Technovit 7200 VLC, Kulzer, Wehrheim, Germany). All specimen was sectioned longitudinally at about 140 µm by a high-precision diamond disc at about 140 µm and ground down to about 35 µm. For each sample, two slides were obtained, and the staining was performed by toluidine blue and acid fuchsin. The bone quality assessment and histomorphometric measurements were performed according to the nomenclature of the American Society of Bone and Mineral Research [20].

The obtained samples were observed in transmitted light by the Nikon microscope ECLIPSE (Nikon, Tokyo, Japan). The bone tissues, medullary space and biomaterial percentages were measured by a histomorphometric analysis software with an image capture tool (NIS-Elements AR 3.0 software, Nikon, Minato, Japan).

## 4. Results

### 4.1. Scaffold Properties 

**Figure 4:**
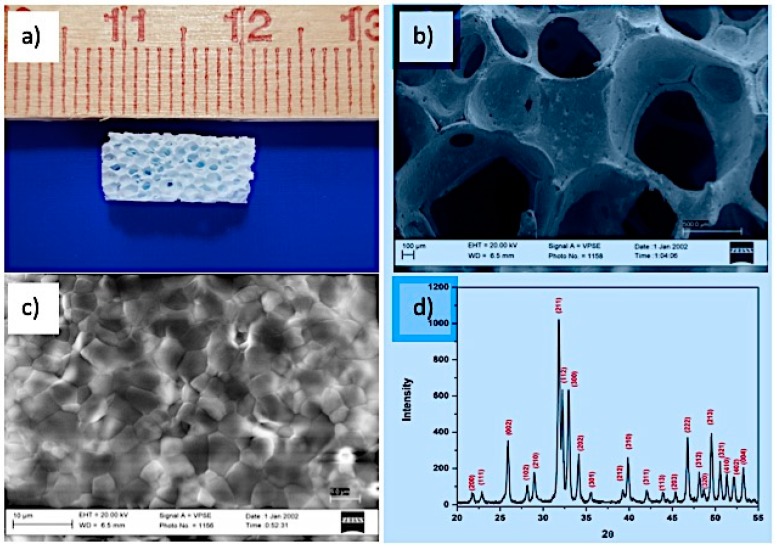
(**a**) A picture of the HA scaffold showing that the sponge replica method was successful in making the HA scaffold, (**b**) the macrostructure of the HA scaffold by SEM reveals that pores are interconnected with an average pore size > 300 microns, (**c**) the microstructure of the HA scaffold by SEM showing a densified structure with well-defined grains having an average grain size < 3 microns without any defects and (**d**) the XRD pattern of the HA scaffold showing a highly crystalline and pure hydroxyapatite phase after sintering the scaffold at 1300 °C.

Figure 4a shows the porous structure of the sponge successfully duplicated to produce the HA scaffold. 

The HA scaffold (Figure 4b) has an open porous structure and highly interconnected pores with a pore size > 300 microns. Such a pore size is a threshold value for the enhanced osteogenesis required for scaffold osteointegration and cell proliferation, as reported by several studies [21,22]. 

The microstructure of the HA scaffold shown in Figure 4c confirms a densified structure with grain boundaries that are well defined and without any noticeable defect. This confirms the good sintering ability of the HA particles. The average grain size of the sintered specimens calculated from the SEM image is around 4 microns. Figure 4d shows the XRD pattern of the HA scaffold after sintering at 1300 °C. The pattern shows a well crystallized form of HA (Joint Committee on Powder Diffraction Standards JCPDS n°09-0432) without the formation of other secondary phases like CaO or TCP. No phase transformation has occurred during the thermal treatment due to the high thermal stability of the starting powder.

The linear shrinkage, porosity, average pore size and compressive strength of the HA scaffold are reported in Table 1. 

The linear shrinkage with 18% sintering is attributed to the closure of pores during the final sintering at 1300 °C. From the scaffold microstructure shown in Figure 4c, a compact polycrystalline material without pores is evidenced. The obtained HA scaffolds have an average porosity of 85% and compressive strength of 0.8 MPa, which is significantly high, providing a suitable and comfortable initial three-dimensional host for cells to build up new bone tissue [12]. 

### 4.2. Clinical and Histological 

No pathological symptoms or signs were recorded at any follow-up clinical visit observation. 

**Figure 5:**
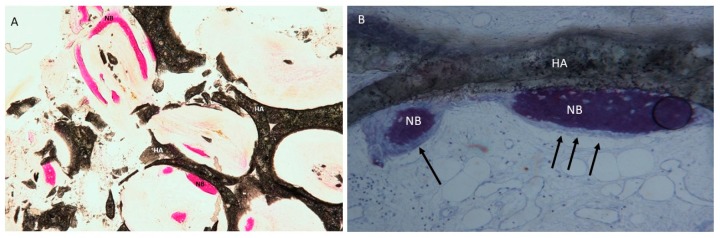
(**A**) A new trabecular bone (NB) was observed inside the interconnected pores of the HA scaffold (HA). Acid fuchsin and toluidine blue staining 10×. (**B**) The new bone (NB) and osteoblasts (arrows) were observed in the internal portion of the block (HA). Toluidine blue staining 100× (× indicates the magnification power of the microscopical observation).

**Figure 6:**
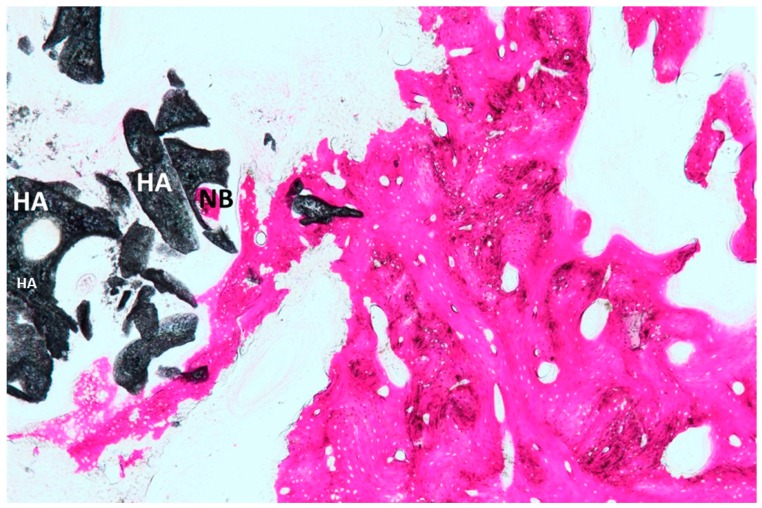
At a higher magnification, we observed a new bone (NB) in direct contact with the HA scaffold (HA). Acid fuchsin and toluidine blue staining 50× (× indicates the magnification power of the microscopical observation).

No acute inflammatory cell infiltrate or foreign body reactions were observed. A newly formed bone with large marrow spaces and wide osteocyte lacunae was present with newly formed vessels (Figure 5 and Figure 6). In a few fields, it was possible to see many osteoblasts in direct contact with the HA block, and a few macrophages cells or osteoclasts were also present. In the histologic analysis, the new bone was evident not only in the external portion of the block (Figure 6) but also in the middle (center) portion of the blocks (Figure 5), inside the highly interconnected pores. In a few fields, it was possible to observe the presence of trabecular bone intensely stained with acid fuchsin, which could be easily differentiated from the neoformed bone. The samples were composed of different tissues, which were 39 ± 1% new bone, 42 ± 3% marrow space, 17 ± 3% residual HA block and 4.02 ± 2% osteoid tissue (Figure 6). Microscopical evidence of new bone formation was also observed in the central portion of the graft block 8 ± 3%.

## 5. Discussion

The objective of this study was to evaluate the bone formation and inflammatory response of an HA scaffold in the internal and external region of the scaffolds. 

The potential of HA porous blocks to repair bone defects in humans is evidenced by the fact that it is able to fulfil the different requirements of bone tissue engineering. Via a sponge replica method, HA porous scaffolds for bone regeneration and augmentation with a desired shape and dimensions are produced. In particular, the outcome of the present study shows that HA porous scaffolds are a highly biocompatible biomaterial [23]. The shrinkage value gives an idea of the dimension of the PU foam for impregnation. A reduction of 18% in size has to be expected before choosing the dimension of the PU foam. No foreign body reaction, inflammatory cell infiltrate or cells were present in any of the specimens. This evidences that the pure and highly crystalline hydroxyapatite phase does not cause any inflammatory reaction. The lack of an inflammatory reaction is related to the absence of calcium oxide produced by the decomposition of HA during sintering or of other contaminants. One of the factors for cell growth and bone formation is the porosity and interconnectivity of the space-making material. Our HA scaffolds shows a high porosity with interconnected pores, which may promote the new bone growth around and inside the scaffolds. 

The histological results show that the HA scaffold is extensively colonized by new bone, evidencing an osteoconductive and osteoinductive attitude. After 3 months, the biomaterial block was found to be non-resorbable. The volume of the residual HA scaffold was 17 ± 3 %, in good accordance with the corresponding porosity of the scaffold (85 ± 3%), which is grafted; furthermore, the data was confirmed by the histologic analysis. These results confirm the findings of other studies, i.e., that pure crystalline HA is non-resorbable. The histologic analysis also confirms that new bone was observed not only in the external portion of the block but also in the internal portion of the blocks. In Figure 6, the HA scaffold structure was intact without breaking any strut, which implies that the mechanical strength of the scaffolds was enough to resist the force exerted when new tissue formed in both the internal and external part of the scaffold. The new bone formation inside evidences that the HA scaffold provides a biocompatible host material and that its interconnected large pores (>300 microns) promote osteoinduction for the bone growth. 

In this study, small cylindrical defects (5 mm) were evaluated and sinus liftings were not. The time chosen for this study was adequate for the dimensions of the bone defects [24,25]. A bone defect with a diameter of 5 mm heals in a shorter time than a maxillary sinus lift [24,26,27,28]. The histomorphometrical evaluation was done following the Piattelli method [19] using ground sections and not decalcified samples [29]. This method has the advantage of not introducing artefacts causing decalcification, with an interference in the interpretation of the results [30]. In this study, the Ha blocks were sterilized with a dry heat treatment because this is one of the best sterilization techniques, able to completely eliminate all viable microorganisms [31]. Steam sterilization was avoided because the presence of water vapor has been shown to cause the hydrolytic degradation of the material to be sterilized [32].

For the treatment of the bone defect of the maxilla and mandible, the autologous bone is still the common therapy. While the autologous bone has provided good results for many years, as it provides osteogenic cells as well as osteoinductive factors, its success is limited by the risk of infection of the donator site, an additional surgical intervention of the donor site and the related postoperative pain [33]. Donor sites for these techniques are intra-oral donor sites and extra-oral donor sites; the extra-oral donor site is chosen when more bone is needed for the treatment of a big bone defect. Some donor sites, such as iliac bone grafts from the anterior site, were associated with a higher mobility and pain intensity than the posterior site [34]. For these reasons, a different biomaterial is proposed for maxillofacial and orthopedic procedures [35], such as synthetic and natural polymers, metallic and ceramic scaffolds, Composite Xenohybrid Scaffolds and biomolecular grafts [36]. Furthermore, injectable, synthetic, bioceramic, etc. biomaterials for the treatment of bone healing have been proposed [37]. Biophysical stimulation is a new technique that uses physical stimuli to treat various diseases in human beings; it can be used in clinical practice, to increase and promote the repair and anabolic activity in tissues, or in association with a drug treatment, to strengthen its activity and lessen side effects [38]. HA bone substitutes are considered to be non-resorbable [39] and are used in a particle and block shape. In fact, the primary mineral component of human bone is hydroxyapatite; several studies have evaluated HA in different clinical situations with a good histomorphometric and histologic result, and this data has been investigated by in-vitro animal and human studies in both dense [40] and porous forms [41]. 

However, most of these studies confirm that HA results in an important graft volume reduction, mostly in sinus lifting. For the preservation of the space-maintenance, the mechanical proprieties of the block graft that is used are important. Different variables influence bone healing; the chemical composition of the biomaterial is one parameter that influences the cellular response, but other factors, such as the porosity and micro-architecture, must be taken into account to achieve better clinical results [42]. 

The limit of the present study is the small sample size, as a result of which comparisons with other studies may prove to be difficult. 

In conclusion, the findings of the present study support that the HA porous scaffold produced by the sponge replica method has an excellent biocompatibility, good mechanical properties for grafting and that it remains intact during new bone formation. It can be an effective solution for the treatment of maxillary bone defects in humans. 

## Figures and Tables

**Table 1:** The linear shrinkage, porosity, pore size and compressive strength of the HA scaffold.

HA Scaffold Physical Properties	
Linear shrinkage (%)	18 ± 1
Porosity %	85 ± 3
Pore size (micron)	>300
Compressive strength (MPa)	0.8 ± 0.1
